# Circadian clock regulation of skeletal muscle growth and repair

**DOI:** 10.12688/f1000research.9076.1

**Published:** 2016-06-30

**Authors:** Somik Chatterjee, Ke Ma

**Affiliations:** 1 Center for Diabetes Research, Department of Medicine, Houston Methodist Research Institute, Houston, TX, 77030, USA; 2 Department of Molecular and Cellular Biology, Baylor College of Medicine, Houston, TX, 77030, USA

**Keywords:** Suprachiasmatic nuclei, Clock Controlled Genes, Myogenic Progenitor Cells, Myosin Heavy Chain

## Abstract

Accumulating evidence indicates that the circadian clock, a transcriptional/translational feedback circuit that generates ~24-hour oscillations in behavior and physiology, is a key temporal regulatory mechanism involved in many important aspects of muscle physiology. Given the clock as an evolutionarily-conserved time-keeping mechanism that synchronizes internal physiology to environmental cues, locomotor activities initiated by skeletal muscle enable entrainment to the light-dark cycles on earth, thus ensuring organismal survival and fitness. Despite the current understanding of the role of molecular clock in preventing age-related sarcopenia, investigations into the underlying molecular pathways that transmit clock signals to the maintenance of skeletal muscle growth and function are only emerging. In the current review, the importance of the muscle clock in maintaining muscle mass during development, repair and aging, together with its contribution to muscle metabolism, will be discussed. Based on our current understandings of how tissue-intrinsic muscle clock functions in the key aspects muscle physiology, interventions targeting the myogenic-modulatory activities of the clock circuit may offer new avenues for prevention and treatment of muscular diseases. Studies of mechanisms underlying circadian clock function and regulation in skeletal muscle warrant continued efforts.

## Abbreviations

SCN: Suprachiasmatic nuclei

CCGs: Clock Controlled Genes

MPCs: Myogenic Progenitor Cells

MyHc: Myosin Heavy Chain

## Introduction

The circadian clock is the overt ~24-hour daily rhythm in physiology and behavior that evolved to respond to earth’s rotation. This evolutionarily-conserved mechanism synchronizes diverse internal biological processes with environmental timing cues to ensure organismal adaptation, fitness and survival ^1– 3^. The circadian clock system consists of a hierarchal organization. The central clock resides in the suprachiasmatic nuclei (SCN) of the hypothalamus and transmits timing signals from light inputs to drive peripheral tissue clocks ^1– 3^. Nearly all tissue/cell types in the body possess cell-autonomous clock circuits that are entrained by central clock signals, but can be fully uncoupled through diet timing manipulations such as restricted feeding ^1, 3– 5^. In recent years, the clock system in skeletal muscle has been recognized to play critical roles in key aspects of skeletal muscle physiology ranging from structural maintenance to functional regulation ^6– 9^. As locomotor activity, the essential function of skeletal muscle in all animal species is under direct circadian clock control through sleep-wake cycles, and the intimate interplay between clock and skeletal muscle physiology is evolutionarily-conserved to ensure fitness and survival. It is therefore possible that the current understandings of the intricate interactions between circadian clock regulation and skeletal muscle at transcriptional, functional and organismal levels are merely at the beginning stages of our endeavor.

## The tissue-intrinsic circadian clock in skeletal muscle

Most physiological processes and diurnal activities of organisms follow distinct daily oscillations, governed via environmental cues by the circadian time-keeping system. This hierarchal machinery is composed of a central pacemaker in the brain’s SCN and peripheral clocks in nearly every tissue and cell types, driven by the central clock pacemaker under normal physiological conditions. The complex interplays between central and peripheral clock systems function in concert to exert proper temporal control on various circadian physiological outputs. At the molecular level, an intricate transcriptional-translational network of circadian clock circuit that generates circadian rhythmicity has been well-defined, although novel modulators of the circadian clock loop continue to emerge. The positive and negative regulators of the molecular clock network are reciprocally regulated through intricate transcriptional and translational feedback loops ^10^. Bmal1 (Brain and Muscle Arnt-like 1) and CLOCK (Circadian Locomotor Output Cycles Kaput), two transcription activators of the molecular clock, form a heterodimer that turns on transcription of its negative regulators. These regulators, Pers (Period1, 2 and 3), Crys (Cryptochrome1 and 2), bind to CLOCK-Bmal1 and inhibit transcriptional activation; whereas the Rev-erbs (Rev-erbα and Rev-erbβ) are direct transcriptional repressors of Bmal1. Notably, Bmal1, the essential transcriptional activator of the molecular clock, is highly expressed in skeletal muscle and initiates target genes transcription through binding canonical E-box, or E’ sequences ^11^. The transcriptional repressors, Rev-erbs, bind to RORE sequence and together with the activator RORα (RAR-related orphan receptor α), generate the circadian oscillatory control of Bmal1 expression. ChIP-sequencing studies in liver have demonstrated extensive overlap of genome-wide cis-acting target promoter sequences between Bmal1 and Rev-erbα/Rev-erbβ ^12, 13^. This suggests that the components of the molecular clock network function coordinately to generate the circadian rhythmicity of their target genes in peripheral tissues, including skeletal muscle. Interestingly, although embryonic stem cells express clock genes, they do not display overt circadian rhythmicity ^14^. The gradual acquisition of diurnal oscillation in clock genes, such as Bmal1 and DBP (D site of albumin promoter binding protein), accompanies their cellular differentiation. This observation raises an intriguing notion of possible coupling between cellular developmental processes with the acquirement of molecular circadian rhythms. Skeletal muscle, the most abundant tissue in mammals that dictates physical activity, possess self-sustaining endogenous molecular clock ^15^.

The circadian clock network plays a prominent role in maintenance of skeletal muscle mass, with the loss of Bmal1 leading to severe sarcopenia with age ^16^. Numerous studies to date involving animal models harboring specific clock gene deletions or mutations have provided useful genetic tools to dissect the roles of the clock circuit in skeletal muscle, as summarized in Table 1. These studies provide strong evidence attesting the importance of circadian clock functions in modulating various aspects of skeletal muscle physiology, including muscle growth and maintenance, contractile performance, structural organization, glucose metabolism and energy production. A remarkable 17% of genes exhibit circadian-like oscillations in skeletal muscle, and nearly 30% of those circadian transcripts lose their rhythmicity in CLOCK-mutant mice ^17^. This indicates that the molecular clock plays a central role in conferring appropriate temporal regulation of clock-controlled genes (CCGs) in skeletal muscle. MyoD1 (Myogenic Differentiation 1), a key transcriptional regulator activated during the early stages of myoblast differentiation and muscle development, has emerged as a CCG based on its distinct circadian expression pattern in adult muscle ^6^. Ablation of core components of the molecular clock, CLOCK or Bmal1, blunts MyoD1 circadian expression as well as its target genes, which is associated with disruption of myofiber sarcomeric organization and muscle contractile function ^6, 17^. Findings of similarly impaired functional deficits in muscle specific force generation in the CLOCK mutant and Bmal1-deficient mice indicate the concerted clock contribution to this essential skeletal muscle function ^6, 16, 18, 19^. Furthermore, Per1, Per2, as well as ROR-deficient mice were found to exhibit related pathologies in muscle structure and function, such as muscle weakness, contractile and locomotor deficits ^6, 20– 22^, further supporting the notion that the clock function is required for skeletal muscle activities. However, so far, the mechanistic link between clock and muscle functional regulation has not been clearly defined.

**Table 1.** Summary of circadian phenotypes and muscle phenotypes observed in mice deficient in various core circadian clock components.

Genotype	Circadian Phenotype	Muscle Phenotype	Reference
Bmal1 (whole body KO)	• Disruption of circadian behavior pattern • Disruption of rhythmic metabolic pattern	• Accelerated aging and reduced life span • Reduced body weight • Reduction in muscle fiber diameter • Fiber-type shift • Decrease in number of mitochondria • Impaired mitochondrial respiration • Altered sarcomeric structure • Impaired muscle regeneration • Reduction in insulin sensitivity • Decrease in glucose oxidation rate	6– 8, 16, 19
Bmal1 (muscle specific rescue in whole body KO)	• Arrhythmic circadian behavior pattern	• Improved life span • Rescued normal body weight • Rescued normal activity levels	19
Bmal1 (muscle specific KO)	• Arrhythmic circadian behavior pattern	• Impaired insulin-dependent glucose uptake • Reduction in glucose oxidation in skeletal muscle • Downregulation of genes involved in glucose utilization • Upregulation of genes involved in lipid metabolism • Shift from a fast to slow fiber-type gene expression • Substrate shift from carbohydrate to lipid utilization, indicative of a more oxidative muscle	69, 70
Bmal2 (constitutive expression rescue in whole body Bmal1 KO)	• Rescue of rhythmic locomotor activity • Rescue of rhythmic metabolic pattern	• Rescue of low body weight • Rescue of rhythmic metabolism	78
CLOCK mutation (whole body)	• Arrhythmic circadian behavior pattern • Loss of rhythmic expression of circadian genes	• Reduction in muscle force • Decrease in exercise tolerance • Disorganized myofilaments • Decrease in number of mitochondria • Abnormal mitochondria • Possible muscle insulin resistance	6, 79
Rev-erb alpha and Rev-erb beta (whole body KO)	• Disruption of rhythmic metabolic pattern	• Impaired muscle maintenance and myogenic differentiation • Lower exercise capacities and impaired exercise endurance • Impaired mitochondrial function and oxidative capacity • Disruption of balance between carbohydrate and lipid metabolism • Induction of muscle autophagy	9, 22, 45, 73, 80
Per1 and Per2 (whole body KO)	• Short circadian period length • Arrhythmic locomotion in constant darkness	• Lower running endurance • Reduced forced locomotor performance • Increased dependence on glycolytic anabolic metabolism • No alteration in skeletal muscle contractile function	20, 21
Cry1 and Cry2 (whole body KO)	• Disruption of rhythmic metabolic pattern	• Glucose intolerant	77
ROR (whole body deletion mutation)	• Slightly longer circadian period length • Disruption of rhythmic metabolic pattern	• Muscle weakness when young • Difficulties in locomotion	75, 76
Dec2/SHARP-1 (overexpression)	• Altered sleep patterns	• Inhibits myogenic differentiation	46, 47
DBP (whole body KO)	• Shorter circadian period length • Less active	• Accelerated aging and shorter life span • Prone to epilepsy	81, 82

Accumulating evidence indicates an intimate interplay between circadian clock machinery and metabolic regulations, either at the level of temporal control evident in many key metabolic processes in distinct metabolic tissues, or in the maintenance of whole-body metabolic homeostasis ^8, 17, 23– 25^. In skeletal muscle, a key organ for metabolic substrate oxidation, nearly 30% of CLOCK-differentially regulated transcripts are involved in metabolism ^17^. Both Bmal1 and Rev-erbα deficiency in mice alters mitochondrial morphology, content or oxidative function ^6, 9^.

So far however, as the majorities of studies of clock function in skeletal muscle are confined to the use to whole-body global ablation models, central clock contribution or secondary effects from other tissues may confound certain findings. Future studies are required to interrogate functions of the intrinsic muscle clock independently of central clock regulation imparted on muscle function. In addition, specific temporal controls conferred by the intrinsic muscle clock may differ in distinct cell types and may be specific to developmental stages. Therefore, there is an urgent need to critically assess the full-range of roles of the intrinsic muscle clock in muscle through developmental stage-selective and tissue or muscle cell type-restricted genetic models.

## Clock modulation of muscle growth, repair and mass maintenance

The first indication that the clock is involved in skeletal muscle maintenance comes from the dramatic phenotype of aging-associated sarcopenia found in Bmal1-null mice ^16^. At 40-weeks of age, the genetic loss of Bmal1 led to a reduction of nearly half of the normal muscle weight with dramatically shortened life span, suggesting a premature aging phenotype in these mice. Interestingly, the lower muscle mass manifests as early as in 8-weeks old mice, when satellite cells are at the peak of their proliferative capacity ^7^. The maintenance of muscle mass encompasses two distinct contributions, one involving myonuclear accretion due to myogenic progenitor proliferation and maturation in early postnatal growth, and mature myofiber hypertrophy in adult stage ^26^. Thus, these findings collectively suggest that the marked reduction of muscle weight in adult Bmal1-null mice may result from the combination of a developmental defect and impaired hypertrophic growth. Furthermore, specific rescue of Bmal1 expression in skeletal muscle was able to prolong survival of Bmal1-null animals, whereas brain-specific rescue was not sufficient ^19^, highlighting that the muscle-intrinsic clock is critical for maintaining proper ambulatory activity that is essential for survival. Miller *et al*. have demonstrated that Bmal1 is required for various aspects essential for proper muscle performance including sarcomeric structure, mitochondrial morphology and muscle contractile activities ^6^, which could be the structural and functional impairments underlying the severe premature aging-like muscle defects observed in Bmal1-deficient animals. Further detailed investigations into the molecular pathways mediating these profound clock effects in skeletal muscle are warranted, particularly in the absence of central clock dysfunction. An intriguing finding is the substantial similarity observed between the sarcomeric disorganization of the Bmal1-null and the CLOCK mutant mice with that of the MyoD-null mutants ^6^. The underlying mechanism linking clock with muscle structure/function regulation could be attributed to the direct transcriptional activation of the Bmal1/CLOCK complex of the identified MyoD1 enhancer element, although non-consensus E-box sequences are involved ^6, 27^. *In vivo*, enhanced expression of the myogenic regulatory factors MyoD1 and myogenin was detected during dark hours, although this diurnal rhythm is strongly suppressed by fasting ^28^. During embryonic development, MyoD1, together with Myf5, specifies the myotome and drives myogenesis ^29, 30^. Thus, this identified specific link of molecular clock with MyoD1 transcription raises an intriguing question as to whether the muscle intrinsic clock participates in muscle development processes or myogenesis. Remarkably, on a genome-wide scale, surveying of CLOCK-controlled mRNA expression in the skeletal muscle reveals that growth, proliferation and differentiation processes comprise a significant 15% of the overall transcripts ^6^. In agreement with this finding, work from our group demonstrated that Bmal1 is a key positive regulator to promote myogenic differentiation ^7^, and its regulation of proliferative behavior and expansion of myogenic progenitor cells is required for tissue regeneration upon injury ^8^. As an evolutionarily-conserved machinery to anticipate and adapt to environmental cues, circadian clock has been implicated in transcriptional control of developmental signaling pathways important for stem cell modulation during tissue remodeling processes ^31– 33^. The clock may provide critical temporal cues to orchestrate the highly ordered stem cell activation, proliferation and differentiation processes required for tissue development, physiological turnover or regenerative repair. The distinct developmental signals required for tissue homeostasis may reflect its specific developmental and functional needs. In skeletal muscle, we found Bmal1 exerts circadian time-dependent transcriptional control on key components of the canonical Wnt signaling pathway ^34^. When tested in muscle injury-elicited regeneration models, including cardiotoxin-induced and freezing injury, mice lacking Bmal1 displayed a significant defect in regenerative myogenic response accompanied by attenuated repair ^8^. Furthermore, the satellite cell expansion process, a major component to ensure proper regeneration, is also impaired due to reduced proliferative capacity. This is likely attributed to Bmal1 regulation of Wnt signaling, since loss of Bmal1 leads to blunted Wnt signaling as observed in Bmal1-null mice muscle regeneration ^6– 8^. Wnt signaling drives embryonic development of the skeletal muscle lineage ^34^, and plays important roles in modulating adult muscle satellite cell functions ^35, 36^. Our original findings provide strong evidence for the cell-autonomous roles of molecular clock in myogenic progenitor cells (MPCs), which provide the major cellular source for muscle growth and repair. This mechanism likely mediates, at least in part, the demonstrated importance of clock function in muscle mass maintenance, particularly during the early postnatal development. Muscle homeostasis and remodeling requires contribution from muscle satellite cells, and their proliferative capacity declines with age. Thus, whether the sarcopenia observed in Bmal1-null mice that resemble early aging could be mediated at least in part by declining clock function in the muscle warrants further investigation. In contrast, as satellite cells are largely not required or necessary for adult skeletal muscle hypertrophic growth ^37^, another possibility is that clock may function in hypertrophic signaling pathways in mature myofibers to contribute to adult muscle mass regulation. These questions could be addressed by muscle developmental-stage specific animal models using currently available genetic tools.

Although the major body of research to date has been focused on the role of Bmal1 as a clock transcription activator, cytosolic Bmal1 was recently identified as a factor facilitating protein translation that links the circadian network and the mTOR (Mechanistic Target of Rapamycin) signaling pathway ^38^. Most intriguingly, the Bmal1-mediated mTOR circadian modulation of translation activities is controlled by daily oscillatory magnesium levels in cells ^39^. These recent findings raise the possibility that Bmal1 and the clock could directly participate in muscle hypertrophic pathways *via* post-transcriptional mechanisms. mTOR signaling, activated by upstream growth factors and PI3 kinase-Akt phosphorylation, is a major regulatory mechanism that promotes protein synthesis to induce skeletal muscle hypertrophy ^26, 40^. In addition, PI3K-Akt-mTOR signaling suppresses muscle atrophy ^40, 41^. Interestingly, multiple components of the Akt/mTOR signaling pathway are reported to be under circadian regulation. Circadian patterns of expression were detected for Akt1 and ribosomal protein S6 of the hypertrophic signaling, and MuRF1 and Fbxo32 within the atrophic pathway in skeletal muscle ^28^. Notably, the circadian profile of Akt1 phosphorylation, an indicator of *in vivo* activity, persists at fasting despite lower levels than ad-libitum feeding, indicating an endogenous rhythm independent of food signals. However, as feeding cycle is dominant zeitgeber for peripheral clocks such as the muscle, there are strong interplays between circadian oscillatory patterns and feeding-fasting switch.

The skeletal muscle phenotypes found in genetic models of additional clock genes further support the notion that the molecular clock as a regulatory circuit exerts profound influence on skeletal muscle mass and function. Both the clock repressor, Rev-erbα, and its reciprocal transcription activator RORα on the RORE responsive element have been implicated in the regulation of myogenic differentiation ^42, 43^. Whereas the constitutive expression of dominant negative Rev-erbα promotes myogenic progression ^42, 44^, myogenic differentiation and myogenic pathways gene expression are suppressed by muscle-specific expression of a truncated RORα mutant ^43^. Importantly, the loss of Rev-erbα deficient mice was found to display lower body weight and altered myosin heavy chain (MyHC) isoform expression with a fast-to-slow MyHC isoform transformation in skeletal muscle, suggesting its involvement in muscle mass maintenance and metabolic control ^45^. The findings of opposing actions of Rev-erbα vs. RORα on myogenic pathways, as well as the opposite effects of clock repressor Rev-erbα vs. activator Bmal1 on myogenesis, strongly suggest orchestration of circadian clock gene functions in regulation of myogenic precursor development. Currently, the molecular mechanisms mediating Rev-erbα vs. RORα actions on myogenesis has not been addressed. Furthermore, based on the significantly increased muscle mass demonstrated in the mPer2-null mice, a potential negative effect of the Bmal1/CLOCK inhibitory regulator, Period 2 (Per2), on muscle growth has been suggested ^21^. Per2 functions in the myogenic cascade remain to be seen. Surprisingly, mPer2 and mPer1 functions in the skeletal muscle are distinct, as the altered muscle mass and metabolic pathways are only evident in the mPer2-null mice but not mPer1-deficient animals. Another transcription inhibitor of CLOCK/Bmal1 function, the basic helix-loop-helix factor Dec2/Sharp1, can suppress myogenic differentiation through its inhibitory interaction with MyoD ^46, 47^.

Taken together, studies of mice harboring genetic mutations of clock genes to date have clearly established a strong link between the molecular clock circuit as a whole and maintenance of skeletal muscle development, growth and potentially hypertrophy. Further studies will be needed to address whether other types of clock disruptions, such as those induced by the dys-synchrony between environmental lighting cycle with endogenous circadian clock cycles, may influence muscle growth and remodeling process. In our post-industrial society, the so-called “social jetlag”, referring to the discordance between our activity/sleep cycle vs. clock cycles, may contribute to the development of certain type of muscle diseases, particularly in the aging population with frequent sleep disorders ^48^. The concerted regulatory functions of the muscle intrinsic clock machinery in maintaining skeletal muscle mass may be important mechanisms to protect against muscle loss in aging-associated or chronic disease-induced muscle wasting conditions. Investigations of underlying molecular pathways mediating clock function in muscle may, therefore, reveal novel therapeutic targets for muscle disease treatment.

## Clock regulation of skeletal muscle structure and function

A major output of circadian clock in animals is its tight control of locomotor activity cycles. As an evolutionarily-conserved mechanism that enables entrainment to the light-dark cycles on earth, the strict behavioral circadian rhythmicity of animals ensures their survival and fitness. Thus, it is not surprising that it has long been recognized that in humans, skeletal muscle torque, strength and power are higher in the late afternoon, between 16:00 and 18:00 hours than compared to the morning ^49– 52, 53^. Major indexes of athletic performance abilities, such as muscle strength, reaction time and flexibilities, display significant time-of-the day dependence ^54, 55^. Knee extensor muscles exhibit a typical diurnal pattern in maximal isometric strength measured in male athletes, which peaks at mid-to-late afternoon period (16:00–20:00 hours) ^56^. Interestingly, partial sleep deprivation was found to have a detrimental effect on the power output of muscle performances, although this effect may depend on the time of the day of the measurements or the onset timing and duration of the sleep disruption ^57, 58^. These findings suggest potentially intimate interplay between clock control, either central or muscle-intrinsic, and physical activity. Most importantly, under various experimental settings, increase in activity level, such as exercise, has been shown to entrain core clock genes and CCGs in humans ^59^ as well as in equine skeletal muscle ^60^. Resistance exercise is capable of shifting expression of diurnally-regulated genes in human skeletal muscle by inducing genes that are normally repressed, while down-regulating genes that are highly expressed ^59^. On the other hand, loss of muscle activity by unilateral sciatic nerve denervation leads to marked atrophy, and reduces the expression of many core clock genes, including Bmal1, Per1, RORα and Rev-erbα in mouse skeletal muscle ^61^. Notably, activity cycles can impact the central clock rhythm. Restricted wheel access in mice, which enforces inverse activity cycles, significantly delays re-entrainment to normal light/dark rhythm ^62^. Together, these studies suggest that physical activity in animals could function as a strong clock entrainment signal, particularly for the skeletal muscle clock. Thus a potential feedback regulatory relationship exists between the circadian clock network and muscle function.

The skeletal muscle circadian transcriptome was first reported by Miller *et al*., based on analysis of gene expression from muscle collected every 4 hours over two circadian cycles ^17^. In skeletal muscle, proteins involved in the regulation of gene transcription are abundant, representing ~17% of rhythmic genes in muscle ^17^. This indicates that many essential functions and physiological processes in skeletal muscle are influenced by the transcriptional output of the clock. Interestingly, a high proportion of cycling transcripts peak midway through the dark phase in mice, coinciding with the peak period of physical activity and feeding in nocturnal species. Particularly, a single large cluster of rhythmic genes displays peak expression at Circadian Time 18 (CT18) of the midpoint of the active phase for mice, even under constant darkness ^17^. However, how much of these processes require central or skeletal muscle-specific molecular clock function has not yet been fully established. Based on the observation that resistance exercise can directly affect expression of key clock components and downstream targets in human skeletal muscle ^59^, the peak expression of rhythmic transcripts in muscle could be attributed to the orchestration of the endogenous muscle clock control and central clock-induced locomotor activity rhythm. Interestingly, although repeated exercise can induce phase-shift of the clock in skeletal muscle, the SCN rhythms are not affected ^15^. Thus, locomotor activity may phase-coordinate the intrinsic rhythmic expression of genes in skeletal muscle with central clock-controlled sleep/wake cycles under normal physiological conditions. These findings together indicate intimate interplays between muscle physical activity and the molecular clock machinery in skeletal muscle, although the underlying mechanistic links, particularly how activity-stimulated signals in muscle is transmitted to clock resetting, phase or amplitude modulation, remain to be elucidated.

## Clock participation in muscle metabolism

The molecular clock machinery governs the temporal control in metabolic processes ^24^. Disruption of this regulatory mechanism profoundly altered metabolic homeostasis leading to the development of obesity and insulin resistance ^63– 67^. Skeletal muscle comprises approximately 40% of the body mass of most mammals, and functions as a major site for glucose disposal and lipid oxidation. Skeletal muscles account for approximately 85% of postprandial insulin-mediated glucose disposal, and changes in muscle function contribute to insulin resistance and metabolic syndromes ^68^. Thus, given its prominent role in temporal control of metabolism, the cell-intrinsic clock machinery in skeletal muscle could be critical for whole-body metabolic homeostasis. There is increasing interest in understanding how the endogenous circadian clock functions to modulate muscle metabolism.

The role of the endogenous skeletal muscle molecular clock in regulating muscle metabolic functions and whole body metabolic homeostasis has emerged recently ^17, 69, 70^. Initial studies of differentially-regulated genes in CLOCK mutants studies indicate that a remarkable ~35% percentage of rhythmic genes in muscle are involved in metabolism ^17^. Further, analysis of circadian metabolic genes revealed a temporal separation of genes involved in substrate utilization vs. storage over a daily period, suggesting a clock-controlled orchestration of distinct catabolic and anabolic metabolic pathways in skeletal muscle ^70^.

To address the contribution of skeletal muscle to whole body circadian energy homeostasis, skeletal muscle-specific Bmal1 deletion was created to test the function of Bmal1 in skeletal muscle glucose metabolism ^69, 70^. Muscle-specific deletions of Bmal1, either constitutively or through inducible-Cre lines, cause impaired insulin-dependent glucose uptake and reduced glucose oxidation in skeletal muscle ^69^. While canonical insulin signaling pathway is not affected, the level of GLUT4 glucose transporter responsible for glucose uptake was significantly lower. It is interesting that these defects in glucose utilization do not lead to overt changes in insulin sensitivity, possibly due to compensatory mechanisms in other tissues. Applying a global gene expression profiling approach in an inducible mouse model of Bmal1 ablation in muscle, a later study revealed significantly altered expression of genes involved in metabolic substrate oxidation ^70^. Significant down-regulation of circadian genes involved in glucose utilization were observed, along with significant up-regulation of genes involved in lipid metabolism. This gene expression profile suggests muscle fiber type switch to a slow oxidative fiber-type consistent with a substrate shift from carbohydrate to lipid utilization, although the precise fiber type distribution in fast or slow muscle fibers were not assessed ^70^. Thus, two independent studies suggest that the endogenous molecular clock may coordinate skeletal muscle metabolic substrate utilization with metabolite availability occurring during fasting-feeding transitions balance, which could play a significant role in whole-body energy partitioning between tissues to maintain metabolic homeostasis ^10^.

The circadian clock repressor gene, Rev-erbα, is known to play important roles in metabolic regulations ^71– 73^. In skeletal muscle, Rev-erbα was found to be highly expressed in oxidative fiber types, and promotes skeletal muscle oxidative capacity through inhibition of mitochondria autophagy and abundance ^9^. A previous study indicated that there was significant fast-to-slow MyHC isoform transformation in Rev-erbα-deficient mice, albeit only in soleus muscle ^45^. Most importantly, as a ligand-dependent nuclear receptor, Rev-erbα is amenable to synthetic ligand modulations. Synthetic agonists of Rev-erbα, display potent anti-obesity and lipid lowering efficacy in mice ^74^. Notably, the activation of Rev-erbα by synthetic agonists induces fatty acid oxidation pathways while suppresses lipid synthesis genes in skeletal muscle, likely a significant contributor to its lipid-lowering effects *in vivo*. In contrast, the exercise endurance of Rev-erbα-deficient mice is reduced, likely a result of lower mitochondrial function in muscle; whereas the activation of Rev-erbα by an agonist improves endurance capacity ^9^. Additional studies of Rev-erbα-deficiency on metabolic homeostasis reveal mild hyperglycemia and increased fatty acid utilization, indicating that Rev-erbα may promote the preferential use of glucose at the expense of peripheral lipid utilization ^73^. These studies establish a foundation to further explore the mechanistic basis of Rev-erbα as a “druggable” target for metabolic diseases, and the potential of modulating the tissue clock circuit as therapeutic strategies. On the other hand, in muscle cells, the dominant negative mutant of RORα, the transcriptional activator of RORE-harboring promoters antagonistic to Rev-erbα, inhibits expression of many genes involved in lipid homeostasis, including carnitine palmitoyltransferase-1 for fatty acid oxidation ^75^. Given that the global loss in the *staggerer* mice leads to reduced muscle strength and hypo-α-lipoproteinemia ^76^, the *in vivo* effects of RORα inhibition in muscle metabolism remains to be seen. In line with findings of the molecular clock regulation in glucose metabolic homeostasis, the loss of Cry1 and Cry2 in mice induces systemic glucose intolerance, although whether this defect is a result of altered muscle glucose disposal needs further detailed studies ^77^.

Taken together, current findings indicate that the clock machinery in skeletal muscle plays a significant role in orchestrating metabolic substrate metabolism. As feeding signals are strong clock entrainment cues, whether clock functions as a temporal mechanism to adapt to feeding-fasting induced metabolic substrate switching remains to be studied. Future investigation into the molecular mechanisms linking clock and muscle metabolic substrate flux may yield novel targets for disease treatment including obesity and diabetes.

## Conclusion

The circadian clock plays key roles in critical aspects of skeletal muscle physiology. Thus, it is imperative to dissect the precise underlying mechanisms involved in these multifaceted interactions. Studies of the intimate interplays of the tissue-intrinsic clock with growth, hypertrophy, activity and metabolism in skeletal muscle would provide a wealth of novel targets for disease prevention or treatment. Particularly, given the importance of the circadian clock network in muscle mass maintenance, interventions targeting myogenic-modulatory activities of the clock circuit may offer new avenues for the prevention and treatment of muscular diseases, particularly those associated with circadian dysregulation.

## Abbreviations

SCN: Suprachiasmatic nuclei

CCGs: Clock Controlled Genes

MPCs: Myogenic Progenitor Cells

MyHc: Myosin Heavy Chain
