# Supplementary material for: Circadian clock regulation of skeletal muscle growth and repair
Source: F1000Res. 2016 Jun 30;5:1549. [Version 1] doi: 10.12688/f1000research.9076.1 (PMC4965692; doi:10.12688/f1000research.9076.1)
Supplement: Peer review history

# Peer review: Referee response for version 1

**DOI:** 10.5256/f1000research.9768.r15134

**Published:** 2016-08-01

**Authors:** Henrik Oster

**Affiliations:** 1 Chronophysiology Group, Medical Department I, University of Lübeck, Lübeck, Germany

This is an interesting review paper summarising the current knowledge about the role of the circadian clock in skeletal muscle development and function.

 I have few comments which might help to improve the paper:

1. Intro: last two sentences are difficult to understand. "As locomotor activity..." this is not a complete sentence / "It is therefore possible..." - consider shortening. Is your main point to say that there is still a lot to do?
2. Chapter "The tissue-intrinsic circadian clock in skeletal muscle"
  - Daily oscillations are not GOVERNED by outside rhythms; consider ENTRAINED
  - Peripheral clocks are not DRIVEN by the SCN; consider COORDINATED/RESET.
3. Carefully check spelling of genes and proteins, in particular capitalisations
4. Consider discussing the role of metabolic feedback on SM clock regulation and function.

I have read this submission. I believe that I have an appropriate level of expertise to confirm that it is of an acceptable scientific standard.

# Peer review: Referee response for version 1

**DOI:** 10.5256/f1000research.9768.r15272

**Published:** 2016-07-28

**Authors:** Lei Yin

**Affiliations:** 1 Department of Molecular & Integrative Physiolog, University of Michigan Medical School, Ann Arbor, MI, USA

The review article summarizes the current understanding about the molecular circadian network for its role in muscle development and muscle biology. This topic is relatively new but will have significant implications in exercise, metabolism and aging. The manuscript is well-written and easy to read. I fully support its indexation in its current format.

 I have a few minor suggestions to help further strengthen the manuscript:

1. It is expected that myocyte-specific molecular clock controls the diurnal expression of key genes that are important for muscle function, such as MyoD. It is less known whether molecular circadian clock within myocytes could directly control the key signalling pathways of muscle metabolism. It would be great if the authors could elaborate on this topic.
2. The question regarding the regulation of muscle circadian clock is missing in the current manuscript. Do we know anything about hormonal or nutritional dependent regulation of muscle circadian clock? Does muscle circadian clock change during obesity, diabetes and the aging process? These knowledge will enhance the readability of this manuscript.

I have read this submission. I believe that I have an appropriate level of expertise to confirm that it is of an acceptable scientific standard.

# Peer review: Referee response for version 1

**DOI:** 10.5256/f1000research.9768.r14726

**Published:** 2016-07-14

**Authors:** R. Daniel Rudic

**Affiliations:** 1 Department of Pharmacology and Toxicology, Vascular Biology Center, Medical College of Georgia, Augusta University, Augusta, GA, USA

Abstract

 Given the clock as IS an evolutionarily-conserved time-keeping mechanism that synchronizes internal physiology to environmental cues, locomotor activities initiated by skeletal muscle enable entrainment to the light-dark cycles on earth, thus ensuring organismal survival and fitness.

*The statement locomotor activities initiated by skeletal muscle is a tenuous statement. Locomotor activities are most likely secondary to central clock function, as was shown in the McDearmon article which showed that muscle specific rescue did not restore locomotor rhythm.*

**Introduction**

 As locomotor activity, the essential function of skeletal muscle in all animal species is under direct circadian clock control through sleep-wake cycles, and the intimate interplay between clock and skeletal muscle physiology is evolutionarily-conserved to ensure fitness and survival.

*Run on sentence*

**The tissue-intrinsic circadian clock in skeletal muscle**

 This hierarchal machinery is composed of a central pacemaker in the brain’s SCN and peripheral clocks in nearly every tissue and cell types, driven by the central clock pacemaker under normal physiological conditions. (repetitive as mentioned in first paragraph)

 ……the brain’s SCN and peripheral clocks in nearly every tissue and cell types

*cell type*

*repetition of ‘intricate’*

 Accumulating evidence indicates an intimate interplay between circadian clock machinery and metabolic regulations, either at the level of temporal control evident in many key metabolic processes in distinct metabolic tissues, or in the maintenance of whole-body metabolic homeostasis

*Overuse of ‘metabolic’ and metabolic regulations should be regulation*

**Clock modulation of muscle growth, repair and mass maintenance**

 as early as in 8-weeks old mice

*8 week*

 prolong survival of Bmal1-null animals, whereas brain-specific rescue was not sufficient

*brain rescue improved survival to 75% in the length of the experiment, saying not sufficient overstated.*

**

*Despite discussion of implications to skeletal muscle disorders, specific links to which skeletal muscle diseases may be implicated are lacking.*

I have read this submission. I believe that I have an appropriate level of expertise to confirm that it is of an acceptable scientific standard.
